# Pediatricians’ Practice Location Choice—Evaluating the Effect of Japan’s 2004 Postgraduate Training Program on the Spatial Distribution of Pediatricians

**DOI:** 10.2188/jea.JE20130117

**Published:** 2014-05-05

**Authors:** Rie Sakai, Günther Fink, Ichiro Kawachi

**Affiliations:** 1 Department of Social and Behavioral Sciences, Harvard School of Public Health, Boston, MA, USA; 2 Department of Global Health and Population, Harvard School of Public Health, Boston, MA, USA; 3 Department of Medical Education, Juntendo University School of Medicine, Tokyo, Japan; 4 Department of Pediatrics and Adolescent Medicine, Juntendo University School of Medicine, Tokyo, Japan

**Keywords:** human resources, physician distribution, postgraduate medical training program, Japan

## Abstract

**Objectives:**

To explore determinants of change in pediatrician supply in Japan, and examine impacts of a 2004 reform of postgraduate medical education on pediatricians’ practice location choice.

**Methods:**

Data were compiled from secondary data sources. The dependent variable was the change in the number of pediatricians at the municipality (“secondary tier of medical care” [STM]) level. To analyze the determinants of pediatrician location choices, we considered the following predictors: initial ratio of pediatricians per 1000 children under five years of age (pediatrician density) and under-5 mortality as measures of local area need, as well as measures of residential quality. Ordinary least-squares regression models were used to estimate the associations. A coefficient equality test was performed to examine differences in predictors before and after 2004. Basic comparisons of pediatrician coverage in the top and bottom 10% of STMs were conducted to assess inequality in pediatrician supply.

**Results:**

Increased supply was inversely associated with baseline pediatrician density both in the *pre-period* and *post-period.* Estimated impact of pediatrician density declined over time (*P* = 0.026), while opposite trends were observed for measures of residential quality. More specifically, urban centers and the SES composite index were positively associated with pediatrician supply for the *post-period*, but no such associations were found for the *pre-period*. Inequality in pediatrician distribution increased substantially after the reform, with the best-served 10% of communities benefitting from five times the pediatrician coverage compared to the least-served 10%.

**Conclusions:**

Residential quality increasingly became a function of location preference rather than public health needs after the reform. New placement schemes should be developed to achieve more equity in access to pediatric care.

## INTRODUCTION

Physicians are a limited health-care resource, and optimizing the distribution of physicians is a major challenge to health systems in many countries. The critical challenge in most settings has been that of recruiting physicians to rural areas, where physician coverage is generally low and child health often significantly poorer compared to urban areas. In this regard, Japan is no exception.

In 2004, the Ministry of Health, Labour and Welfare (MHLW) in Japan initiated a new postgraduate medical education program to improve the quality of residency training. Prior to 2004, most of the graduating medical students in Japan underwent postgraduate training at a hospital affiliated with the university from which they graduated,^1^ with university hospitals systematically placing medical residents in affiliated rural facilities under their supervision. In contrast, the new program allowed residents to choose their training location directly through a national matching system, thereby reducing university hospitals’ ability to dispatch recent graduates to their rural, affiliated training hospitals.

While the reform has had a major impact on physicians’ placement, research on the impact of the new system on physician distribution in Japan has been limited.^2^^–^^4^ In this study, we analyze the impact of the policy change on the spatial distribution of pediatricians. We focus on pediatricians because the Japanese government has highlighted pediatrics as one of the specialties experiencing a serious shortage,^5^^,^^6^ fully recognizing the direct implications of pediatrician coverage for child health.^7^ Our study’s objectives are threefold: first, to investigate the principal determinants of pediatricians’ practice-location choice; second, to examine differences in pediatrician supply before and after the launch of the new program in 2004; and third, to examine changes in the distribution of pediatrician coverage since 2004.

## DATA AND METHODS

### Unit of analysis

Our study is an ecological analysis of physician supply and distribution. The Japanese government is organized into three layers of administration: municipal, prefectural, and national. Japanese prefectures and municipalities correspond roughly to states and counties in the United States (though Japan does not operate on a federalist system like the United States). Japan has 47 prefectures. During the study period, Japan underwent administrative re-organization through a large-scale merging of municipalities. The total number of municipalities decreased from 3232 to 1750 between 1998 and 2010. All the data were adjusted for the new municipal boundaries by merging former smaller municipalities into larger ones. The main unit of analysis used for this study is the “secondary tier of medical care” (STM), which typically comprises several municipalities. Each prefecture is divided into four to ten STMs on the basis of its medical resources, transportation, and geography. STMs are roughly comparable to Hospital Service Areas in the U.S. There were 356 STMs in Japan at the time of this study, which are generally considered independent administrative areas from a health service perspective, and are less prone to local spillovers than municipalities or counties which have been used in other studies.^2^^,^^4^^,^^8^^–^^12^ The total number of STMs did not change significantly, although their borders were redrawn. The boundaries of STMs in 2004 were used in this study.

### Data

#### Dependent variable

Our main variables of interest were differences in numbers of pediatricians between the two 4-year time periods: between 1998 and 2002, which represents the period before the 2004 program started (*pre-period*), and between 2006 and 2010, which represents the period after the 2004 program started (*post-period*).

#### Independent variables

Two types of measures were considered as the main predictors of interest for the pediatrician supply analysis: measures of need, and measures of residential quality as generally highlighted in the literature on physicians’ practice-location choice.^13^^–^^16^ The two primary measures of need we use are pediatricians per 1000 children under the age of 5 (pediatrician density) and under-5 mortality at the beginning of the two periods.

As a first measure of residential quality, urban/rural status was considered. Municipalities were divided into five categories based on the metropolitan area code defined by the Ministry of Internal Affairs and Communications: 1) central cities of major metropolitan areas, 2) central cities of metropolitan areas, 3) surrounding municipalities of central cities of major metropolitan areas, 4) surrounding municipalities of central cities of metropolitan areas, and 5) other municipalities. This classification is revised based on the results of the national census conducted every five years.

The classifications from 2000 and 2005 were used to analyze the *pre-period* (1998–2002) and *post-period* (2006–2010), respectively. In this study, major metropolitan and metropolitan areas were combined into one category, since there were only five central cities of metropolitan areas among 1750 municipalities in 2000 and six in 2005. This resulted in three basic groups: 1) STMs that include central cities for major metropolitan areas and metropolitan areas, which are defined as urban centers (*n* = 26 in *pre-period* and *n* = 28 in *post-period*); 2) STMs that include surrounding municipalities of central cities of major metropolitan areas and metropolitan areas, which are defined as suburban areas (*n* = 131 in *pre-period* and *n* = 134 in *post-period*); and 3) others, which are defined as rural (*n* = 199 in *pre-period* and *n* = 194 in *post-period*). Rural is used as a reference group in the models. Because no standard definition of the term “rural” exists,^17^^–^^20^ we checked the robustness of the model with respect to alternative urban/rural measures. Previous studies have employed one of the following definitions^17^: 1) metropolitan statistical area,^9^^,^^19^ which is comparable to metropolitan area codes in Japan; 2) population size^10^^,^^17^^,^^19^; and 3) population density.^17^^,^^21^ We employed population density to define urban/rural status as an alternative definition. Under this alternative definition, the STMs with a population density of more than 1000/km^2^ are defined as urban (*n* = 67 in *pre-period* and *n* = 66 in *post-period*), and the remaining are defined as rural (*n* = 289 in *pre-period* and *n* = 290 in *post-period*).

As an additional measure of local residential quality, we also included a composite index of socioeconomic indicators (SES composite index), which was created from socioeconomic variables for education, occupation, and income to avoid multicollinearity. The index was based on a factor analysis of the percentage of the population with a college-level education, the percentage of white-collar workers, the unemployment rate, and per capita income. Factor scores, formulated by a principal component analysis with varimax rotation, were used to construct a composite index to represent each aspect of socioeconomic status for the study units.

Table 1 describes each of the variables selected in the models. In addition to measures of needs and residential quality, measures of professional interactions were considered in this study because the published literature has identified that professional interactions were important factors that affect physicians’ decisions regarding their practice locations.^13^^–^^16^ Data (not including temperature and humidity data, which were only available at the prefecture level) were obtained at the municipality level and aggregated by STM. In addition to the independent variables described in Table 1, we created two dichotomous indicators designated *closedummy* and *opendummy*. *Closedummy* equaled 1 if STMs had children’s hospitals that closed during the study period and equaled 0 otherwise. *Opendummy* equaled 1 if STMs had children’s hospitals that were newly opened during the study period and 0 otherwise. For example, in 2010, three children’s hospitals in Tokyo were closed to merge into one large children’s hospital with 561 beds. *Closedummy* and *opendummy* were created to adjust for this special event.

**Table 1.** Variables selected in the models

Variable	Explanation
Measures of need	
Under 5-year-old mortality	The number of deaths under the age of five per number of births
Pediatrician density	The number of pediatricians per 1000 children under the age of 5^a^
Measures of residential quality	
Urban/rural status 1) urban centers, 2) suburban areas, 3) rural areas	The metropolitan area code defined by the Ministry of Internal Affairs and Communications
Per capita income	
Percent of the population with a university-level education	As a proxy for educational level in the community
Unemployment rate	The number of unemployed individuals per the number of all individuals currently in the labor force (workforce)
Percent of white-collar workers	The number of professionals, technical workers and managers, and administrators per number of workforce
Primary school students per number of primary schools	As a proxy for children’s educational opportunities
Crime rate	The number of crime per total population as a proxy for neighborhood safety
Temperature	As a proxy for climate discomfort. The discomfort index was calculated by using temperature and humidity and used in the model.
Humidity	
Measures of professional interaction	
The density of physicians other than pediatricians	The total number of physicians excepting pediatricians per 1000 in a population older than 15 years old^b^
Hospital beds per 1000 population	
The presence or absence of children’s hospitals	
The presence or absence of medical schools	As a proxy for continuing education

^a^This age group is used to calculate pediatrician density because the population in this age group tends to have a greater demand for pediatric medical services.^13^

^b^This age group is used to calculate physician density because pediatricians typically treat children under 14 years of age.

### Data sources

We obtained data from multiple sources. Data for the total number of physicians and pediatricians were obtained from the Survey of Physicians, Dentists, and Pharmacologists,^22^ which is conducted every two years by the MHLW. All licensed physicians are expected to complete this survey and register their working addresses and specialties under the Medical Practitioners Law.^23^ The estimated registration rate is reported to be between 87% and 90%.^24^ The definition of primary care in Japan is ambiguous, and there is no standard specialty term or professional organization that corresponds to the family physician in the U.S. or to the general practitioner in the U.K.,^25^ so pediatricians play the dominant role in pediatric care in Japan. We did not include general practitioners or family physicians in this study, although these specialties do provide pediatric care in other countries.^26^ The local population of children younger than 5 years old was obtained from the Basic Resident Registers^27^ and was used to calculate pediatrician-to-population and physician-to-population ratios.

Factors previously shown to be associated with physician supply^13^^–^^16^ were obtained from publicly available secondary data. Numbers of births and deaths for children under 5 years old were obtained from the vital registration system.^28^^–^^31^ The oldest data yielding under-5 mortality dates back to 1999, so the under-5-year-old mortality of 1999 was applied in the analysis of the period 1998–2002. The data for the following five variables were obtained from *Regional Statistics by Municipalities*, which was produced by the Ministry of Internal Affairs and Communications (MIAC)^32^: 1) per capita income; 2) number of hospital beds; 3) number of primary schools; 4) number of primary school students; and 5) crime rates, defined as number of crimes per 1000 population. The data for the following four variables were obtained from the Japanese Census^33^^–^^35^: 1) Metropolitan area codes; 2) percentage of the population with a college-level education; 3) unemployment rate; and 4) percentage of white-collar workers. The Japanese Census is conducted every five years. Unemployment rates and the percentage of white-collar workers were calculated using the mean of 1995 and 2000 data and applied to the time period 1998–2002. Corresponding data from 2005 were applied to analysis of the time period 2006–2010. The percentage of the population with a college-level education is only collected every ten years. The data from 1990 was no longer publicly available. Therefore, data from 2000 was applied to the time period 1998–2002. The mean of 2000 and 2010 data was applied to the time period 2006–2010.

To assess average climate (as a potential factor in physician location preference), the discomfort index, which was developed at the U.S. Weather Bureau (currently the National Weather Service) and has been widely used in previous studies,^36^^,^^37^ was calculated by using temperature and humidity. Temperature and humidity could only be obtained at the prefecture level; these data were obtained from *Regional Statistics by Prefectures*, which was produced by MIAC^38^ and used in the model.

### Statistical analysis

Descriptive statistics of all variables were presented as means with standard deviations and 95% confidence intervals (CIs) for the period 1998–2002 (*pre-period*) and for 2006–2010 (*post-period*). Mean equality tests were performed to examine the statistical significance of the observed differences.

Ordinary least-squares regression models were used to analyze changes in STM-level pediatrician supply in the *pre-period* and the *post-period*. For both periods, changes in pediatrician supply were evaluated as a function of STM-level baseline factors, which were defined as 1998 conditions for the *pre-period* and as 2006 conditions for the *post-period*. A test of coefficient equality in regressions was performed to examine significant differences in coefficients between the *pre-period* and the *post-period*.

Finally, we investigated the changes in relative access to pediatricians over time. To detect any trends towards greater regional disparities in pediatrician supply, we created a baseline by determining which STMs fell into either the top 10% or bottom 10% in pediatrician coverage at the first interval in the study (1998–2000). We then tracked the coverage rates for these two STM subgroups, using two-year intervals for the period 1998–2010.

A two-tailed *P*-value of less than 0.05 was considered statistically significant. All analyses were performed using SAS software 9.2 (SAS Institute, Inc., Cary, NC, USA).

## RESULTS

Figure shows the distribution of the change in the number of pediatricians in each STM before (1998–2002) and after (2006–2010) the 2004 reform. Only 17% of STMs had no change over the 4-year horizon analyzed (*n* = 62 in *pre-period*, and *n* = 59 in *post-period*).

**Figure.**
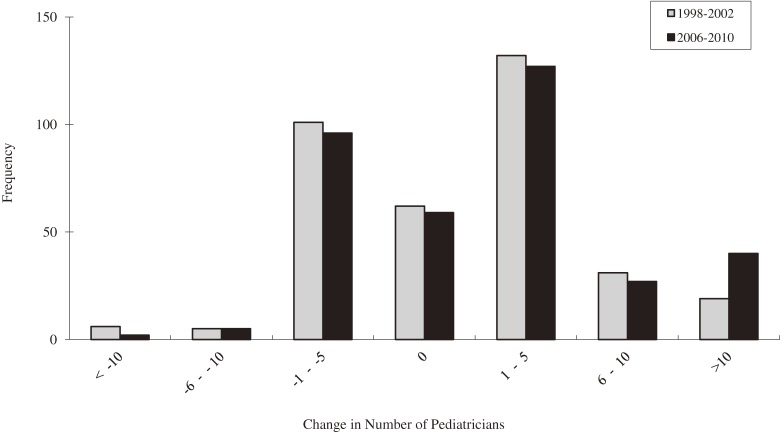
Distribution of change in the number of pediatricians before (1998–2002) and after (2006–2010) the reform.

Table 2 shows the aggregate level change in pediatrician supply from 1998 to 2010 at the national level as well as stratified by urban/rural status. At the national level, while the child population gradually declined over the period, the pediatrician supply increased substantially: from 1998–2002, there was a 3.5% increase in the absolute number of pediatricians, and from 2006–2010, there was an 8% increase. The changes in pediatrician density (pediatricians per child population) were even larger, with a 4.2% increase in the *pre-period* and an 11.5% increase in the *post-period*. Stratified analysis showed that relative changes in the number of pediatricians and pediatrician density were the largest in rural areas and the smallest in urban centers in the *pre-period*. However, in the *post-period*, the opposite effect was observed: the smallest changes were in rural areas and the largest in urban centers. The relative changes in pediatrician density were more than 10% in all areas; however, the increase in pediatrician density in rural areas was mainly due to decline in pediatric populations rather than increase in pediatrician supply. More specifically, the 11.46% increase in pediatrician density in urban centers was the result of a large increase in the number of pediatricians (11.58%) and a very small increase (0.10%) in pediatric population, while the 11.30% increase in pediatrician density in rural areas was the result of a moderate increase in the number of pediatricians (3.59%) and a larger decrease in pediatric population (−6.93%).

**Table 2.** The aggregate level change in pediatrician supply at the national level

	1998	2002	4-year relative change (%) (2002–1998) /1998	2006	2010	4-year relative change (%) (2010–2006) /2006
National level						
Number of pediatricians	13 989	14 481	3.50	14 700	15 870	8.00
Under 5 year old population	5 938 861	5 865 028	−1.20	5 569 073	5 383 149	−3.30
Pediatrician density^a^	2.4	2.5	4.20	2.6	2.9	11.50
Urban centers						
Number of STMs^b^	26			28		
Number of pediatricians	4941	5017	1.54	5278	5889	11.58
Under 5 year old population	1 593 511	1 606 922	0.84	1 628 803	1 630 509	0.10
Pediatrician density^a^	3.10	3.12	0.69	3.24	3.61	11.46
Sub-urban areas						
Number of STMs^b^	131			134		
Number of pediatricians	4905	5109	4.16	5356	5769	7.71
Under 5 year old population	2 464 260	2 452 980	−0.46	2 369 592	2 290 763	−3.33
Pediatrician density^a^	1.99	2.08	4.64	2.26	2.52	11.42
Rural areas						
Number of STMs^b^	199			194		
Number of pediatricians	4143	4355	5.12	4066	4212	3.59
Under 5 year old population	1 881 090	1 805 126	−4.04	1 570 678	1 461 877	−6.93
Pediatrician density^a^	2.20	2.41	9.54	2.59	2.88	11.30

^a^Pediatricians per 1000 children under the age of 5.

^b^Secondary tiers of medical care.

Table 3 shows descriptive statistics of dependent and independent variables for the *pre-period* and *post-period*, along with the *P-value* resulting from testing the Equality of Means. Pediatrician and physician densities increased (both *P* < 0.001) and under-5 mortality decreased (*P* < 0.001) significantly. It is worth highlighting that most socioeconomic measures (per capita income, unemployment rate, and composite SES Index) deteriorated significantly over time, a reflection of Japan’s poor economic performance over the 2005–2010 period.

**Table 3.** Descriptive statistics of all dependent and independent variables: the secondary tier of medical care as a unit of analysis

	1998–2002			2006–2010			*P value*^c^
	
	Mean	SD^a^	95% CI^b^	Mean	SD^a^	95% CI^b^	
Number of pediatricians	39.99	(55.98)	[35.87 to 44.11]	42.94	(61.08)	[38.44 to 47.43]	0.3423
Pediatrician density^d^	2.14	(1.13)	[2.06 to 2.22]	2.49	(1.17)	[2.41 to 2.58]	<0.0001
Under-5 mortality	4.61	(2.11)	[4.45 to 4.76]	3.65	(2.34)	[3.48 to 3.82]	<0.0001
Per capita income (‘000)^e^	13.50	(3.31)	[13.25 to 13.74]	12.45	(3.44)	[12.20 to 12.71]	<0.0001
Percent of the population with a college-level education	10.66	(5.16)	[10.12 to 11.20]	11.76	(5.29)	[11.21 to 12.32]	0.0048
Unemployment rate	4.05	(1.23)	[3.96 to 4.14]	6.14	(1.51)	[6.03 to 6.25]	<0.0001
Percent of white-collar workers	14.37	(2.41)	[14.19 to 14.55]	13.93	(2.23)	[13.69 to 14.16]	0.0031
SES composite Index^g^	0.05	(1.00)	[−0.02 to 0.13]	−0.05	(1.00)	[−0.13 to 0.02]	0.0404
Number of primary students/school	270.90	(135.3)	[261.00 to 280.90]	265.00	(138.9)	[254.8 to 275.2]	0.4178
Crime rate	1.45	(0.72)	[1.40 to 1.50]	1.09	(0.52)	[1.05 to 1.13]	<0.0001
Temperature (°C)	15.82	(2.53)	[15.3 to 16.34]	15.59	(2.36)	[15.10 to 16.07]	0.5169
Humidity (%)	70.28	(4.70)	[69.31 to 71.25]	69.44	(4.36)	[68.54 to 70.33]	0.2047
Discomfort Index^h^	60.05	(3.87)	[59.25 to 60.84]	59.68	(3.58)	[58.94 to 60.41]	0.4997
Physician density^i^	1.88	(0.86)	[1.81 to 1.94]	2.01	(0.92)	[1.94 to 2.07]	0.0065
Hospital beds per 1000 population	13.91	(4.91)	[13.55 to 14.27]	13.99	(4.77)	[13.64 to 14.34]	0.7745

^a^Standard deviation.

^b^Confidence intervals.

^c^*P value* of mean equality test.

^d^Number of pediatricians per 1000 children under the age of 5.

^e^Japanese yen was converted into US$ using the rate that applied in March 2013 of approximately 95 Japanese yen per USD.

^f^The percent of the population with a college-level education is only collected every ten years. Therefore, data from 2000 were used in both models.

^g^A composite index of socioeconomic indicators created from the percent of the population with a college-level education, percent of white-collar workers, the unemployment rate, and per capita income.

^h^Calculated by using temperature and humidity.

^i^The total number of physicians excepting pediatricians per 1000 in a population older than 15 years old.

Tables 4-1 and 4-2 show the correlation coefficients between outcomes and the predictors of interest in the *pre-period* (Table 4-1) and *post-period* (Table 4-2).

**Table 4-1.** Correlation coefficient between outcome and predictors of interest in *pre-period* (1998–2002)

	Difference in number of pediatricians between 1998 and 2002	Under 5 year old mortality	Pediatrician density	Urban centers	Sub-urban areas	SES composite Index
Difference in number of pediatricians between 1998 and 2002	1					
Under 5 year old mortality	−0.010	1				
Pediatrician density	−0.089	−0.049	1			
Urban centers	0.067	−0.035	0.384**	1		
Sub-urban areas	0.021	−0.066	−0.093	−0.214**	1	
SES composite Index	0.120*	−0.142**	0.504**	0.447**	0.259**	1

*P < 0.05, **P < 0.01.

**Table 4-2.** Correlation coefficient between outcome and predictors of interest in *post-period* (2006–2010)

	Difference in number of pediatricians between 2006 and 2010	Under 5 year old mortality	Pediatrician density	Urban centers	Sub-urban areas	SES composite Index
Difference in number of pediatricians between 2006 and 2010	1					
Under 5 year old mortality	−0.040	1				
Pediatrician density	0.242**	−0.091	1			
Urban centers	0.538**	−0.046	0.314**	1		
Sub-urban areas	−0.016	0.035	−0.135*	−0.227**	1	
SES composite Index	0.552**	−0.103	0.460**	0.461**	0.234**	1

*P < 0.05, **P < 0.01.

Table 5-1 presents the results from the multivariate regression analyses. Pediatrician supply was inversely associated with baseline pediatrician density both in the *pre-period* (*P* < 0.001) and *post-period* (*P* = 0.004). However, the estimated impact of pediatrician density declined from −3.068 (95% CI −4.197 to −1.940) to −1.386 (95% CI −2.333 to −0.440) over time (*P* = 0.026). Opposite trends were observed for measures of residential quality, as urban centers and the SES composite index showed statistically positive associations with pediatrician supply in the *post-period* (*P* < 0.001 for urban center, and *P* = 0.001 for SES index), whereas no such effects were found for the *pre-period*. This suggests that residential quality emerged as a driving force in pediatricians’ location preference following the 2004 legislation. The coefficient equality test showed significant differences in coefficients between the *pre-period* and *post-period* both for urban centers (*P* < 0.001) and the SES composite index (*P* = 0.047).

**Table 5-1.** Results of multivariate regression models^a^

Main predictors of interest	1998–2002				2006–2010				*P value* of coefficient equality test
	
	Estimated coefficient	SE^b^	95% CI^c^	*P value*	Estimated coefficient	SE^b^	95% CI^c^	*P value*	
Measures of Child Care Need									
Under 5 year old mortality	0.037	0.159	[−0.275 to 0.348]	0.82	0.056	0.150	[−0.237 to 0.350]	0.71	0.93
Pediatrician density^d^	−3.068	0.576	[−4.197 to −1.940]	<0.001	−1.386	0.483	[−2.333 to −0.440]	0.004	0.026
Measures of Residential Quality									
Urban centers	−3.068	1.654	[−6.310 to 0.174]	0.06	11.31	1.714	[7.902 to 14.725]	<0.001	<0.001
Sub-urban areas	−1.476	0.818	[−3.079 to 0.126]	0.07	−1.394	0.891	[−3.141 to 0.353]	0.12	0.95
Rural areas		Reference				Reference			
SES composite Index^e^	0.562	0.560	[−0.536 to 1.660]	0.32	2.291	0.662	[0.994 to 3.587]	0.001	0.047

^a^The models included the control variables: number of primary school students per number of primary schools, crime rate, discomfort index calculated by temperature and humidity, the density of physicians other than pediatricians, hospital beds per 1000 population, the presence or absence of children’s hospitals, the presence or absence of medical schools, *closedummy* and *opendummy*. *Closedummy* equaled 1 if the secondary tiers of medical care (STMs) had children’s hospitals that closed during the study period and equaled 0 otherwise. *Opendummy* equaled 1 if STMs had children’s hospitals that were newly opened during the study period and 0 otherwise.

^b^Standard Error.

^c^Confidence Intervals.

^d^Ratio of the number of pediatricians to the under-5-year-old population.

^e^SES composite index was created from the percent of the population with a college-level education, percent of white-collar workers, the unemployment rate, and per capita income.

When considering the national average association, we estimated that each unit increase in pediatrician density (one pediatrician per 1000 children under the age of 5) in 1998 and in 2006 was associated with a decrease in the number of pediatricians of 3.07 in 2002 and of 1.39 in 2010, after adjustment for all other variables. As for residential quality, we estimated that urban centers gained 11.31 more pediatricians from 2006 to 2010 compared to rural areas, and that each unit increase in the SES composite index in 2006 was associated with an increase in the number of pediatricians of 2.29 from 2006 to 2010, after adjusting for all other variables.

Table 5-2 shows the result of a robustness check using population density as an alternate urban/rural definition. Results were similar under this definition.

**Table 5-2.** Results of multivariate regression models^a^

Main predictors of interest	1998–2002				2006–2010				*P value* of coefficient equality test
	
	Estimated coefficient	SE^b^	95% CI^c^	*P value*	Estimated coefficient	SE^b^	95% CI^c^	*P value*	
Measures of Child Care Need									
Under-5 mortality	0.051	0.16	[−0.263 to 0.365]	0.75	0.063	0.161	[0.254 to 0.379]	0.699	0.96
Pediatrician density^d^	−3.041	0.579	[−4.175 to −1.907]	<0.0001	−1.349	0.521	[−2.370 to −0.329]	0.01	0.030
Measures of Residential Quality									
Urban areas	−1.4091	1.183	[−3.727 to 0.908]	0.23	2.943	1.449	[0.104 to 5.783]	0.042	0.021
Rural areas		Reference				Reference			
SES composite index^e^	0.2791	0.5543	[−0.807 to 1.365]	0.62	2.955	0.708	[1.568 to 4.342]	<0.0001	0.031

^a^The models included the control variables: number of primary school students per number of primary schools, crime rate, discomfort index calculated by temperature and humidity, the density of physicians other than pediatricians, hospital beds per 1000 population, the presence or absence of children’s hospitals, the presence or absence of medical schools, *closedummy* and *opendummy*. *Closedummy* equaled 1 if the secondary tiers of medical care (STMs) had children’s hospitals that closed during the study period and equaled 0 otherwise. *Opendummy* equaled 1 if STMs had children’s hospitals that were newly opened during the study period and 0 otherwise.

^b^Standard Error.

^c^Confidence Intervals.

^d^Ratio of number of pediatricians to under 5 year old population.

^e^SES composite index was created from the percent of the population with a college-level education, percent of white-collar workers, the unemployment rate, and per capita income.

Table 6 shows pediatrician supply for the best-supplied top 10% of STMs, as well as the least-supplied bottom 10% of STMs in two-year increments from 1998 to 2010. Pediatrician density gradually increased both in the top and bottom 10% of STMs over the period. It is clear that this was due to decrease in child population rather than due to increase in the number of pediatricians. Inequalities were rather large, with the best-served areas benefitting from coverage levels averaging five times higher than least-served areas. Rather remarkably, coverage inequalities gradually declined from 1998–2002, but have been increasing ever since.

**Table 6.** Pediatrician supply of the top 10% and bottom 10% of the secondary tiers of medical care (STMs)

	1998	2000	2002	2004	2006	2008	2010
Top 10% (*n* = 35)							
# of pediatricians^a^	3528	3492	3683	3187	3301	3529	3437
Under 5 yo population^b^	839 073	824 143	869 086	710 850	714 212	719 794	644 954
Pediatrician density^c^	4.20	4.24	4.24	4.48	4.62	4.90	5.33

Bottom 10% (*n* = 35)							
# of pediatricians^a^	183	209	245	233	242	180	187
Under 5 yo population^b^	228 196	239 110	252 337	234 704	234 768	172 223	174 469
Pediatrician density^c^	0.80	0.87	0.97	0.99	1.03	1.05	1.07

Ratio^d^	5.24	4.85	4.36	4.52	4.48	4.69	4.97
Difference^e^	3.40	3.36	3.27	3.49	3.59	3.86	4.26

^a^Number of pediatricians.

^b^Population under the age of five.

^c^Number of pediatricians per population under the age of five.

^d^Ratio in pediatrician density (Top 10%/Bottom 10%).

^e^Difference in pediatrician density (Top 10% − Bottom 10%).

## DISCUSSION

Our study explored community factors that affected changes in pediatrician supply at the community level, as well as the impact of the 2004 national training program on the pediatrician supply.

Our results suggest that the supply of pediatricians to underserved areas has increased overall during the study period. However, this occurred not as a result of improved placement, but rather as a result of declining pediatric populations, with a consequent increased overall supply of physicians. When looking at the relative supply of pediatricians, increasing inequalities are noticeable after 2004, with a substantially larger fraction of doctors choosing practice locations in urban areas with superior living conditions and local health needs declining in relative importance as a factor driving the location preference of pediatricians.

As noted by Pearl,^39^ physicians can clearly not be socially and economically set apart from the rest of society. In Japan, population growth rates between 1998 and 2002 and between 2006 and 2010 were 0.7% and 0.002%, respectively, while those in population sizes of the top largest 10 cities were 2.1% and 3.3%, respectively.^27^ These data suggest that pediatricians’ preference to move to urban areas is reflected by broader movements in the general population. However, the results of this study also suggest that the 2004 medical training scheme had the unintended consequence of making it easier for new medical graduates to choose their location for training, which exacerbated regional inequalities in physician supply.

Previous studies^39^^–^^42^ have found a positive relationship between economic variables and physician supply. Freed et al.^40^ noted that a portion of health care services can be considered “luxury” consumption goods on which people spend a higher percentage of their money as their incomes rise, and consequently, states having a higher gross domestic product per capita can provide greater employment and economic opportunities for physicians. Chang et al.^41^ pointed out that pediatricians in the pre-managed-care era may have had more incentives to settle where individuals could afford routine health care maintenance services such as immunizations, which were not covered by some insurance plans. In our study, we note that while the regional SES composite index was not a significant driver of pediatricians’ practice location choices in the *pre-period*, it became significant in the *post-period*.

In 2007, the Japanese cabinet decided to raise the number of medical school admissions for the first time in 23 years in order to increase the number of physicians.^43^ Since then, the number of medical school admissions has increased from 7626 in 2007 to 8923 in 2011.^44^ The government has stated that they will continue to raise the number of admissions until 2019.^44^ In 2015, the biggest cohort ever will be newly certified as physicians,^43^ and will go into postgraduate medical training programs. Although pediatrician coverage will inevitably increase as the number of pediatricians rises (while the pediatric population continues its inexorable decline), new placement schemes will have to be developed to achieve equity in pediatric care in the long run.

There are some limitations to consider in interpreting the results of this study. First, publicly available data do not include data on whether a physician works full time or part time. This analysis was based on an overall headcount, which might overestimate the number of pediatricians.

Second, publicly available data do not include information on physician age or gender. Therefore, we were unable to analyze trends by these data, although previous studies^16^^,^^45^^–^^47^ noted the effect of gender or age on differences in physicians’ practice location choices. Previous studies variably found that younger physicians were more prone to change their practice location,^16^^,^^45^^,^^47^ that female physicians tended to practice in the same state as that in which they received their graduate medical education,^46^ or that female physicians tended to move.^16^^,^^45^ The effects of gender are still unclear, with a report from Canada stating that gender is not a significant predictor of the probability of a physician moving to another province.^47^ The differences observed in our study could, in theory, be attributable to age or gender differences in the composition of the pediatric work force in the *pre-* and *post-periods*, respectively. In the aggregate, the percentage of female physicians in pediatrics increased from 29.3% in 1998 to 33.0% in 2010, while the percentage of pediatricians in their 20s and 30s decreased from 34.5% in the *pre-period* to 32.1% in 2010. However, assuming that both groups display urban preferences due to differences in schooling and other infrastructure needs, the net bias generated by these two factors is likely small.

Third, some local governments have their own polices to attract or send physicians to particular (generally underserved) areas, and these policies could affect physician practice location choice. Two policies that possibly affect pediatrician distribution are worth mentioning: 1) local efforts to increase the concentration of pediatricians in regional pediatric centers; and 2) local policies to send more physicians to rural areas with major coverage shortages.

In 2005, the government proposed a guideline prescribing the concentration of the pediatrician workforce at regional pediatric centers. The move was made in order to utilize pediatricians efficiently, as well as to prevent burnout.^48^ To our knowledge, there is only very limited information on regions that accomplished the targeted regional concentrations in centers. Conceptually, this should be a relatively minor issue; however, the purpose of this paper is to analyze the distribution of pediatricians across STMs, while the framework for the pediatric healthcare system as established by the Japan Pediatric Society^49^ mostly defines the concentration of the pediatrician workforce within each STM.^50^^,^^51^

Of greater concern in our setting are regional efforts to attract more physicians to rural areas where the shortage is most severe. The policy most frequently adopted at present is a program which reimburses medical school tuition to those students who agree to work in the rural areas to which they are assigned for a designated number of years after graduation. This policy was first instituted on a large scale in 2006.^52^ The first graduates covered by this policy graduated in 2012; therefore, this policy does not impact the results of our study. A similar, but much older, program exists within Jichi Medical University, a medical school co-funded by all prefectures to send their medical graduates to the areas with a shortage of physicians; however, this school was established in 1972, and thus should not affect any differences observed between the *pre-period* and the *post-period* either.

It is possible that a number of regions adopted pro-rural policies at the municipality level. For example, some regions use public funding to provide a better salary to physicians who work in rural areas to ensure physician supply in underserved areas.^53^ To the extent that these efforts may have increased in the *post-period*, the estimated coefficients reported in this paper would underestimate the true effect of the policy change on pediatrician practice location choices.

Fourth, the survey of Physicians, Dentists, and Pharmacologists was revised in 2006. The new category of “Residents” was added. The data of “1998–2002” included the number of medical residents, but the data of “2006–2010” did not. Since the primary dependent variable is the change in the number of pediatricians, which should only be affected marginally by the relatively constant supply of residents in the *pre-period*, the 2006 revision in the survey should not greatly affect the results.

Last, we are only able to comment on whether the community factors driving pediatrician practice location differed before and after 2004. Regrettably, we are unable to establish whether the new training program was the primary cause of changes in pediatric practice location choices (*ie, this difference in trends might have happened even without the 2004 reform, and we might have observed natural trends in pediatrician supply*). Nor can we determine whether the changes impacted actual population health status.

Despite these limitations, we believe that our study contributes to the debate regarding the inequality in pediatrician supply for the following reasons. First, in previous research on the distribution of physicians, the units of analysis used coincided with the municipal level in Japan and the county level in the U.S.^2^^,^^4^^,^^8^^–^^12^ However, physician visits may involve crossing county borders because of location or travel route considerations.^54^ Our study accounted for both geographic location and travel route conditions by using STMs that accounted for these points. Second, we used two different definitions of urban/rural status because no standard definition of the term “rural” exists,^17^^–^^20^ and both analyses showed similar results.

Finally, this study also introduced a new methodology in workforce analysis, with the use of differences in the number of pediatricians between two time points to capture the direction of changes in pediatrician supply, whereas the Gini coefficient^4^^,^^8^^,^^10^^,^^12^^,^^55^ has been widely used in previous studies to assess the distribution of physicians.

### Conclusions

Over the past 15 years, pediatrician supply in Japan has increased substantially, while residential choice has increasingly become a function of practice location preferences rather than public health needs. New placement schemes will need to be developed to achieve more equity in access to pediatric care.
